# A conductive polymer nanowire including functional quantum dots generated via pulsed laser irradiation for high-sensitivity sensor applications

**DOI:** 10.1038/s41598-021-90460-8

**Published:** 2021-05-27

**Authors:** Michiko Sasaki, Masahiro Goto

**Affiliations:** International Center for Materials Nanoarchitectonics (MANA), National Institute for Materials Science, 1-2-1 Sengen, Tsukuba, Ibaraki 305-0047 Japan

**Keywords:** Polymers, Polymers

## Abstract

The fabrication of functional conductive polymer nanowires (CPNWs), including ultrahigh-sensitive flexible nanosensors has attracted considerable attention in field of the Internet of Things. However, the controllable and space-selective growth of CPNWs remains challenging, and a novel synthetic technique is required. Herein, we demonstrate the synthesis of space-selective CPNWs that include quantum dots (QDs) with changeable optical properties via single-pulse laser irradiation in air at atmospheric pressure. Time-resolved shadowgraphy was applied to monitor the synthetic process of the CPNWs and optimise the process conditions. The electrical conductivity of the CPNWs with QDs (QD-CPNWs) was analysed in the presence and absence of light irradiation and was found to change drastically (over six times) under light irradiation. QD-CPNW synthesis under laser irradiation shows great potential for fabricating highly photosensitive functional nanomaterials and is expected to be applied in the production of ultrahigh-sensitive photosensors in the future.

## Introduction

Polymer-based nanowires (PNWs) are an important class of one-dimensional nanostructures. Given their flexibility, stretchability and low cost together with their excellent electrical, magnetic and optical properties, PNWs show great potential as the basic building blocks of nanosensors^1–16^, nanoelectronic devices^17–31^ and solar cells^32–35^ formed via bottom-up fabrication. In particular, conductive PNWs have attracted considerable attention because of their tunable electrical and optical properties in molecular structures or as chemical dopants. Nanosensors and nanoelectronic devices involving PNWs require space-selective fixing, easy drying process, low cost, size control and property control. Several approaches for the synthesis of PNWs have been reported, including the wetting of porous alumina templates^36^, electrospinning^37^ and methods based on solution chemistry. However, these methods do not provide space selectivity and size controllability, and the types of PNWs that can be synthesized are restricted by the requirement of high temperatures and/or chemical solvents. Therefore, the ability to synthesize controllable PNWs for nanosensors and nanoelectronic devices is currently limited.

A new method for the synthesis of PNWs using pulsed laser irradiation was reported in 2009^38^. In this low-cost approach, the PNWs are generated at the laser focus point of the polymer source film with space selectively under dry conditions; iron oxide nanoparticles were also successfully introduced into the PNWs, demonstrating the potential of this method for modulating the PNW properties. However, only the synthesis of nonconductive PNWs was reported.

In this work, we demonstrate the space-selective synthesis of conductive polymer nanowires that include quantum dots (QD-CPNWs) with changeable electrical properties via single-pulse laser irradiation in air at atmospheric pressure. Time-resolved shadowgraphy was used to monitor the synthetic process and optimize the process conditions. The electrical properties and current–voltage (*I*–*V*) characteristics of the QD-CPNWs were measured using a scanning electron microscopy (SEM)-based nano-probe system in the presence and absence of light irradiation. The conductivity of the QD-CPNWs was drastically increased by approximately six times by light irradiation.

## Results and discussion

**Figure 1:**
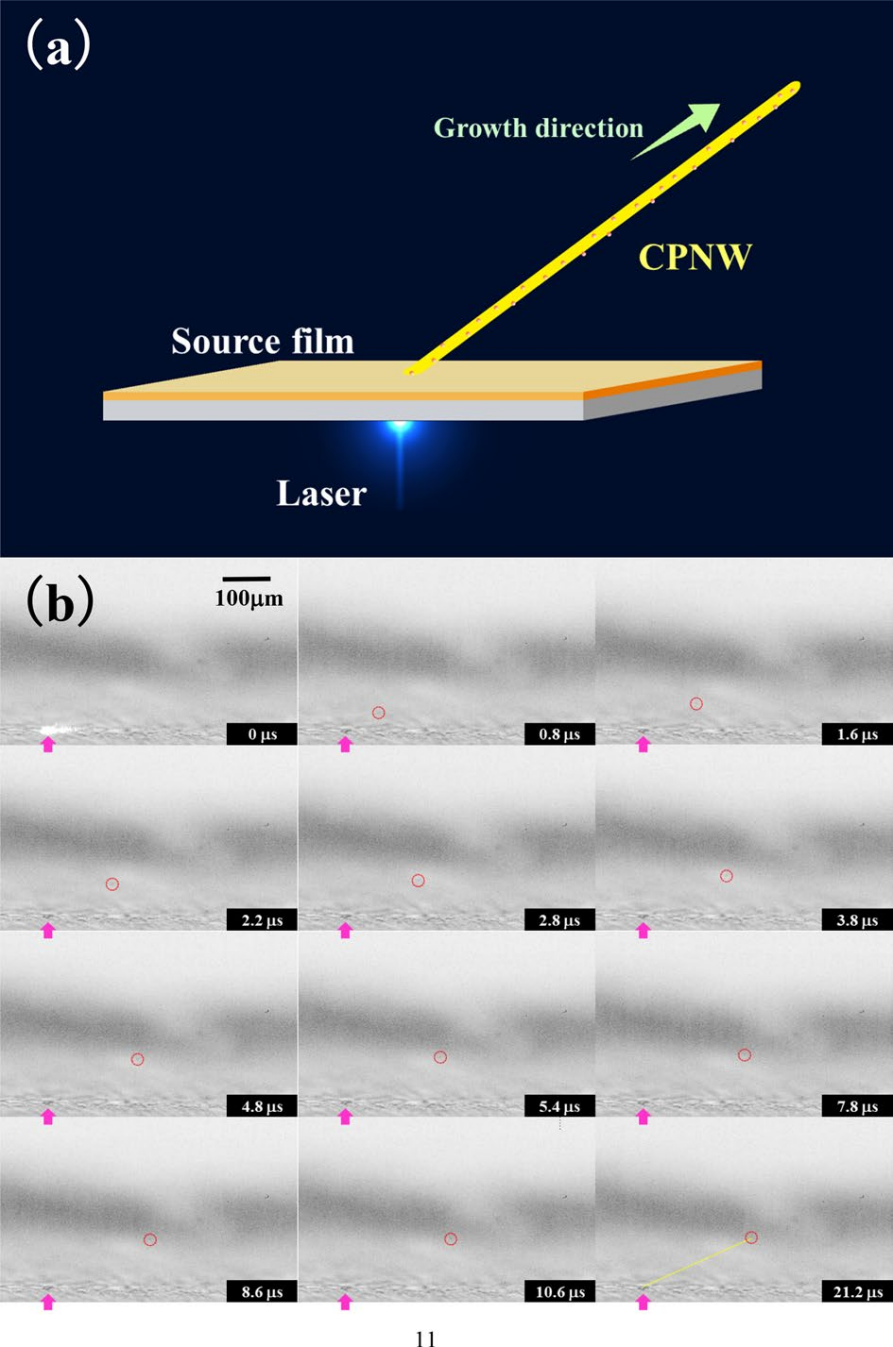
(**a**) Conductive polymer nanowire (CPNW) growth process via pulsed laser irradiation. The CPNW including quantum dots (QDs) is diagonally grown from the laser irradiation point on a source film. (**b**) Time-resolved shadowgraphy images of a PEDOT-PEG PNW including CdSe/ZnS core–shell QDs (QD-CPNW). The laser irradiation point and head of the CPNW are indicated by yellow and pink arrows, respectively.

Inspired by the concept of PNWs synthesized via pulsed laser irradiation, we successfully synthesized poly(3,4-ethylenedioxythiophene)-block-poly(ethylene glycol) (PEDOT-PEG) CPNWs including CdSe/ZnS core–shell QDs via single-shot pulsed laser irradiation at a wavelength of 440 nm, pulse duration of 800 ps and laser fluence of 145–155 J/cm^2^. The QD-CPNW growth process via pulsed laser irradiation is shown in Fig. 1a. Briefly, the QD-CPNWs were generated by a single pulsed laser irradiation from a source film prepared by drop-casting a solution of CdSe/ZnS core–shell QDs dispersed in PEDOT-PEG propylenecarbonate solution on a glass substrate. Figure 1b shows the time-resolved shadowgraphy images of the PEDOT-PEG CPNWs including CdSe/ZnS core–shell QDs (hereafter referred to as QD-CPNWs). The laser irradiation point and the head of the QD-CPNW in Fig. 1b are indicated by yellow and pink arrows, respectively. The main body of the QD-CPNW was difficult to detect from the shadowgraphy images because the QD-CPNW diameter was approximately 40 nm, which exceeds the diffraction limit of the irradiated flush lamp light. Fortunately, it was easier to detect the head of the QD-CPNW. The growth rate of the QD-CPNW after the beginning of irradiation 1 µs was 99 µm/s, and the growth process was terminated within 10 µs. The QD-CPNW grew diagonally from the source substrate. (see Supplementary Movie 1). This explains why the CPNWs could not be detected for a long time, because it can only be detected the growth direction of the CPNW was nearly parallel to the shadowgraphy screen surface. In contrast, nonconductive PNWs grew perpendicular to the surface and remained in the standing state for more than a few hours.

**Figure 2:**
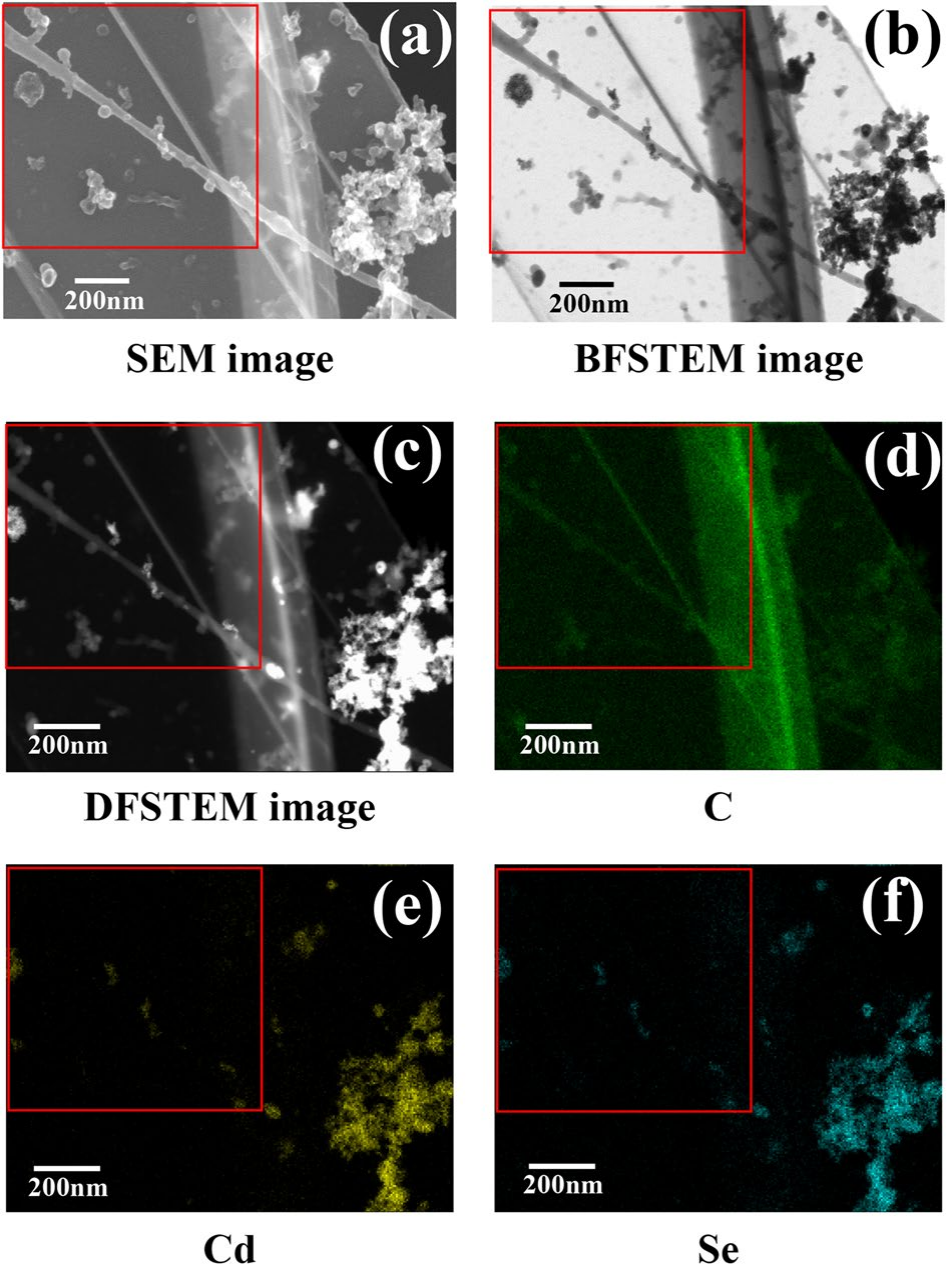
(**a**) High-resolution scanning electron microscopy (SEM), (**b**) bright-field scanning transmission electron microscopy (BFSTEM), (**c**) dark-field scanning transmission electron microscopy (DFSTEM), and energy-dispersive X-ray spectroscopy (EDX) maps of (**d**) carbon, (**e**) Cd and (**f**) Se for the QD-CPNWs.

High-resolution SEM, bright-field scanning transmission electron microscopy (BFSTEM), dark-field scanning transmission electron microscopy (DFSTEM), and energy-dispersive X-ray spectroscopy (EDX) images of a PEDOT-PEG PNW including CdSe/ZnS core–shell QDs (diameter = approximately 6.3 nm) are shown in Fig. 2. The conductive PEDOT-PEG QD-CPNW can be observed in the SEM image (Fig. 2a), as confirmed by the EDX carbon map (Fig. 2d), and the QD-CPNW morphology was determined. Long CPNWs with lengths of approximately 260 µm and diameters of approximately 40 nm were generated. The CdSe/ZnS core–shell QDs were adhered to or included in the CPNWs, as indicated by the SEM, BFSTEM and DFSTEM images in Fig. 2a–c, respectively. Bright spots corresponding to relatively heavy elements were detected in the CPNWs. The CdSe/ZnS core–shell QDs were confirmed by EDX to contain Cd (Fig. 2e) and Se (Fig. 2f) elements, and the signal positions of these elements corresponded to the QD locations in the SEM images.

The basic growth mechanism of the QD-CPNWs was similar to that previously reported for nonconductive PNWs^38,39^. The source film was first photo-excited by the pulsed laser. The PEDOT-PEG polymer was then heated by the thermal energy converted from the absorbed laser light and melted. The melted polymer together with the CdSe/ZnS core–shell QDs was ejected from the surface of the source film, resulting in QD-CPNW growth. The typical length of the QD-CPNW was approximately several hundred µm, shorter than that of the nonconductive PNWs. As mentioned before, the growth direction of the conductive and nonconductive PNWs was diagonal and perpendicular to the source substrate, respectively. To generate QD-CPNWs, the laser fluence should be optimized; the range of optimum laser fluence for QD-CPNW growth in this study was narrow. The appropriate laser fluence for QD-CPNW growth was determined to be approximately 145–155 J/cm^2^. For CPNW without QDs, a higher laser fluence of approximately 160–170 J/cm^2^ was required for growth. It was demonstrated that the QDs assisted the absorption of laser light necessary for growth.

**Figure 3:**
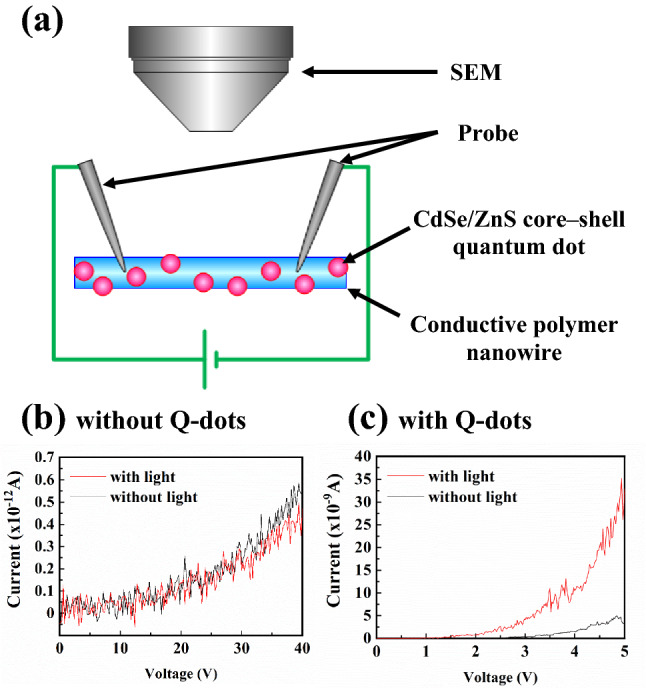
(**a**) The measuring process of a CPNW conductivity using a SEM-based nano-probe system. (**b**) *I*–*V* curves of the CPNWs without QDs in the presence and absence of light irradiation, (**c**) with QDs.

The electrical characteristics of the QD-CPNWs were analysed using a fine-structure evaluation system (Fig. 3a). The *I*–*V* curves of the CPNWs without QDs in the presence and absence of light irradiation are shown in Fig. 3b. The CPNWs were irradiated using a light-emitting diode (LED) at a fixed irradiance of 30 µW/cm^2^. The electrical properties were not changed by light irradiation in this case. However, the *I*–*V* properties were drastically changed when the CPNWs included QDs, as shown in Fig. 3c; the current value was enhanced more than nine times under light irradiation at an induced potential of 4.94 V. The conductivities of QD-CPNWs without and with light irradiation were determined to be 0.045 and 0.42 S/m, respectively, at 4.94 V. The conductivity of QD-CPNWs without light irradiation decreased to 1.1 × 10^−5^ S/m for CPNW (without QD). This may result from changes in the polymer network structure following the introduction of QDs; however, there is currently no possibility to analyze this. The QD-CPNW could be detected weak intensity light as the changing the conductivity.

**Figure 4:**
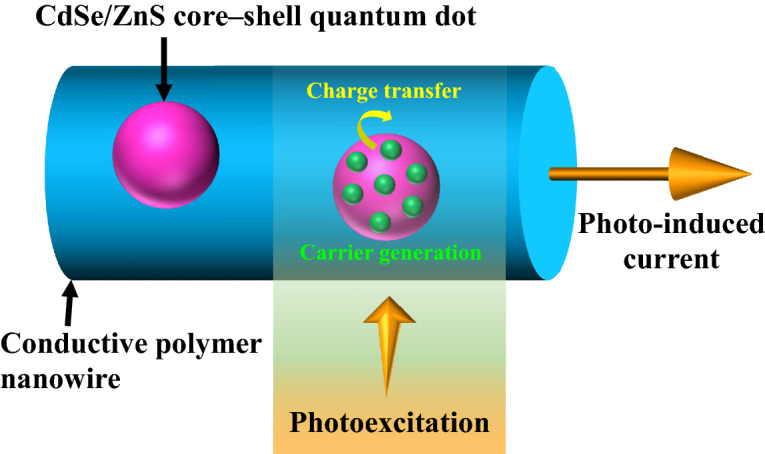
A generation model of the photo-induced current of the QD-CPNW. Carriers such as electrons and holes are generated efficiently in the QDs under weak light irradiation, and charge transfer from the QDs to CPNW occurs, which is observed as the photo-induced current.

Figure 4 shows a model of the photo-induced current of the QD-CPNW. Carriers such as electrons and holes are confined in the QDs in a state close to each other^40,41^, causing carrier-to-carrier interactions such as the Auger effect and carrier amplification. Therefore, the carriers are generated efficiently in the QDs under weak light irradiation, and the QDs cause the charge transfer to CPNW, which is observed as the photo-induced current. Photosensivity (R) is evaluated using the following equation.

$$ {\text{R}} = {\text{I}}_{{{\text{ph}}}} {\text{/P}} $$

where I_ph_ is photocurrent (I_ph_ = I_light_ – I_dark_) and P is light power. The QD-CPNW exhibited a high R value of ~ 1.6 × 10^5^ A/W at an induced potential of 4.94 V, which is among the highest values observed for two-dimensional optoelectronic materials. For example, the R values of B-doped Si-QD/graphene phototransistors^42^ and WSe_2_–In_2_O_3_ NW phototransistors^43^ exhibited values of ~ 10^9^ (UV-NIR), ~ 44.9 (MIR, 77 K), and 7.5 × 10^5^ (637 nm), 3.5 × 10^4^ (940 nm). The R value shown by QD-CPNW in this work is comparable to that of the highest value optoelectronic materials.

## Conclusions

QD-CPNWs were successfully obtained via pulsed laser irradiation. The structure of a QD-CPNW was analysed in detail by STEM-EDX. CdSe/ZnS core–shell QDs were dispersed in the PEDOT-PEG matrix of the PNW. The growth mechanism of the QD-CPNWs was proposed. The electrical characteristics of the QD-CPNWs in the presence and absence of light irradiation were analysed. The conductivity of the QD-CPNWs was drastically increased by light irradiation because of the carrier generation in the QDs, in contrast to the CPNWs without QDs. The proposed method for synthesizing QD-CPNWs via laser irradiation can be applied to produce highly photosensitive functional nanomaterials. This method is expected to be useful for the generation of ultrahigh-sensitivity photosensors in the future.

## Methods

### Conductive polymer nanowire fabrication

**Figure 5:**
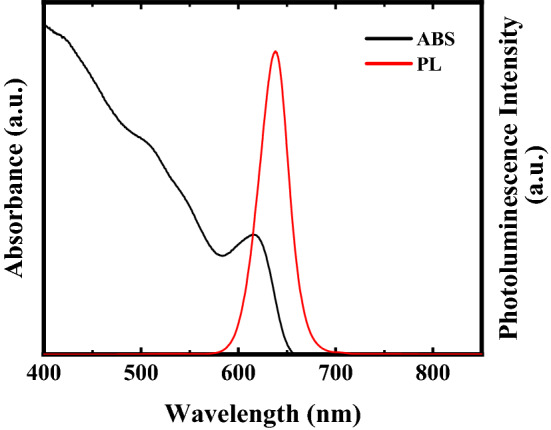
Absorption and photoluminescence spectra of CdSe/ZnS core–shell QDs.

A mixed solvent was obtained by mixing 15 ml of a solution of PEDOT:PEG (8 wt. %) dispersed propylenecarbonate (Sigma-Aldrich) and 5 ml of CdSe/ZnS QDs (diameter = approximately 6.3 nm, fluorescence center wavelength = 640 nm, 5 mg/ml solution in toluene; Lumidot, Sigma-Aldrich 694,606). The QDs consisted of CdSe cores covered with thin ZnS shells (CdSe/ZnS core–shell QDs). Typical absorption and photoluminescence spectra of the QDs in toluene is shown in Fig. 5. The size of the CdSe/ZnS core–shell QDs with 6.3 nm in diameter was selected considering the photo absorption property which absorbed the emission wavelength range of white LED.

Ofcourse, the appropriate QDs diameter can be selected according to the requirement of the application. To create the source films, 2-ml drops of solvent were cast on borosilicate glass substrates and then dried in atmosphere in a clean booth for 30 min. To synthesize QD-CPNWs, the source films were irradiated by a focused, single-pulse laser (wavelength = 440 nm, pulse duration = 900 ps) through an objective lens with a 20 × magnification. The diameter of the laser focus spot was approximately 1.1 µm. We attempted to grow QD-CPNWs) in the laser irradiation area of the source film surface.

### Characterization and photo-induced current measurement

**Figure 6:**
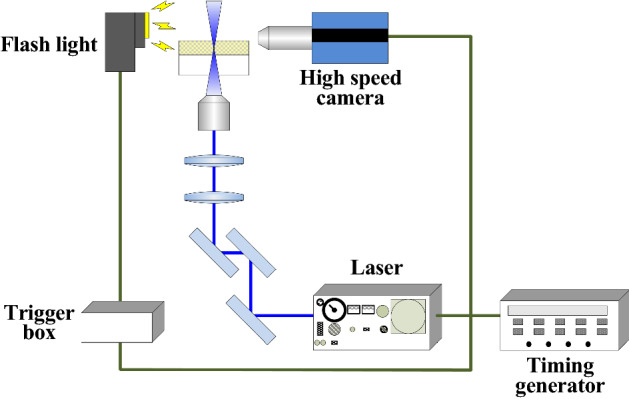
Experimental shadowgraphy setup used to monitor QD-CPNW growth using a high-speed camera. QD-CPNW was generated from the polymer film surface by laser irradiation through the borosilicate glass substrate. The high-speed video camera recorded the shape of the QD-CPNW as a shadowgraphy image. A timing generator was used for setting the time delays for the QD-CPNW generation laser, the flashlight, and high-speed video camera recording.

The QD-CPNW growth process was monitored by shadowgraphy using an inverted optical microscope (IX-70, Olympus Co.) equipped with a high-speed video camera (HyperVision HPV-2A, Shimadzu Co., Ltd.) (Fig. 6). The QD-CPNW was generated from the source polymer film surface by laser irradiation through the transparent borosilicate glass substrate. The flashlight from the opposite side of the high-speed video camera illuminated the generated QD-CPNW. The high-speed video camera recorded the shape of QD-CPNW as the shadowgraphy video. A timing generator was used for setting the time delays for the QD-CPNW generation laser, the flashlight, and high-speed video camera recording. The diameter of the QD-CPNW exceeded the optical diffraction limit while the resolution of obtained video flame images reached the limit. In addition, although a high-intensity flash lamp was used for high-speed video shadowgraphy, the video image contrast of the QD-CPNW was also limited.

The shape and composition of a QD-CPNW was analysed by an ultrahigh-resolution SEM (SU9000, Hitachi High-Technologies Corp.). The carbon microgrid for transmission electron microscopy (TEM) observation was placed on the source film to capture the grown QD-CPNWs. In order to increase the amount of captured QD-CPNWs, the laser fluence was increased to a value greater than that of the fluence. Therefore, the captured QD-CPNWs on the carbon microgrid were decomposed to small fragments. We mounted the QD-CPNWs on a carbon microgrid for transmission electron microscopy (TEM) observation. The electrical conductivity of the synthesised QD-CPNWs was analysed using a fine-structure device characteristic evaluation system (N-6000, Hitachi High-Technologies Corp.). The *I*–*V* curves of the QD-CPNWs analysed by this system to confirm reproducibility, together with the *I*–*V* curve data of different samples, are shown in Supplementary Fig. S1.

We used a white LED to assess the change in QD-CPNW conductivity under light. The fluence of the LED was measured using a photodiode sensor (PD300-UV, Ophir Corp.). The relative spectral power distribution of the LED are shown in Supplementary Figure S2.

## Supplementary Information

Supplementary Video 1.

Supplementary Information 1.

Supplementary Information 2.

Supplementary Information 3.
