# Supplementary figures and images for: A conductive polymer nanowire including functional quantum dots generated via pulsed laser irradiation for high-sensitivity sensor applications
Source: Sci Rep. 2021 May 27;11:11203. doi: 10.1038/s41598-021-90460-8 (PMC8159946; doi:10.1038/s41598-021-90460-8)

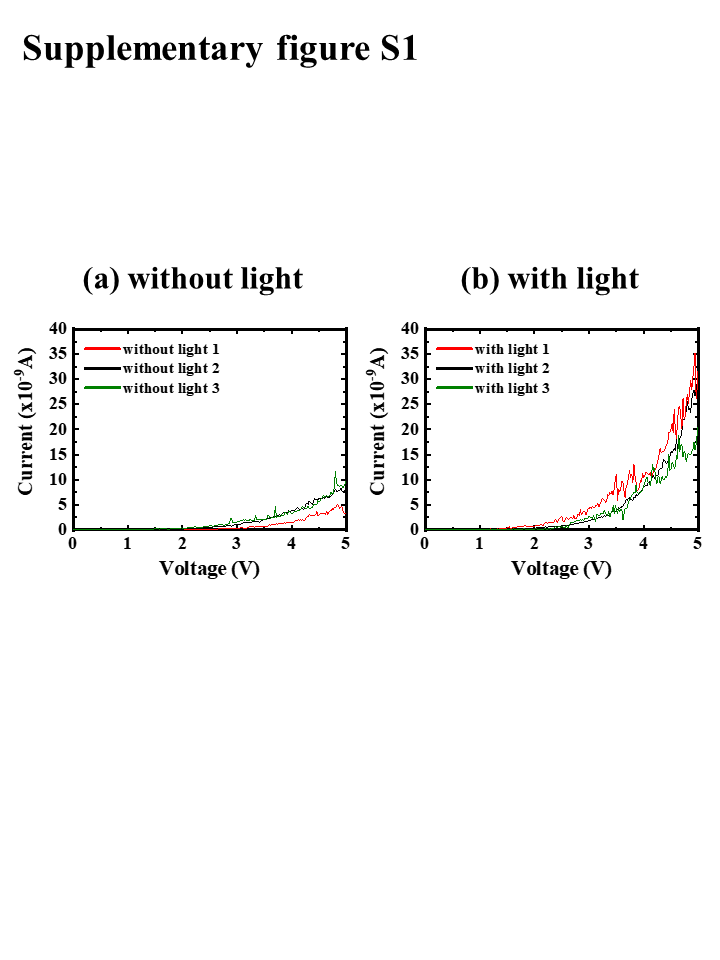

Supplement: Supplementary file 2 — Supplementary Information 1. [file 41598_2021_90460_MOESM2_ESM.tif]

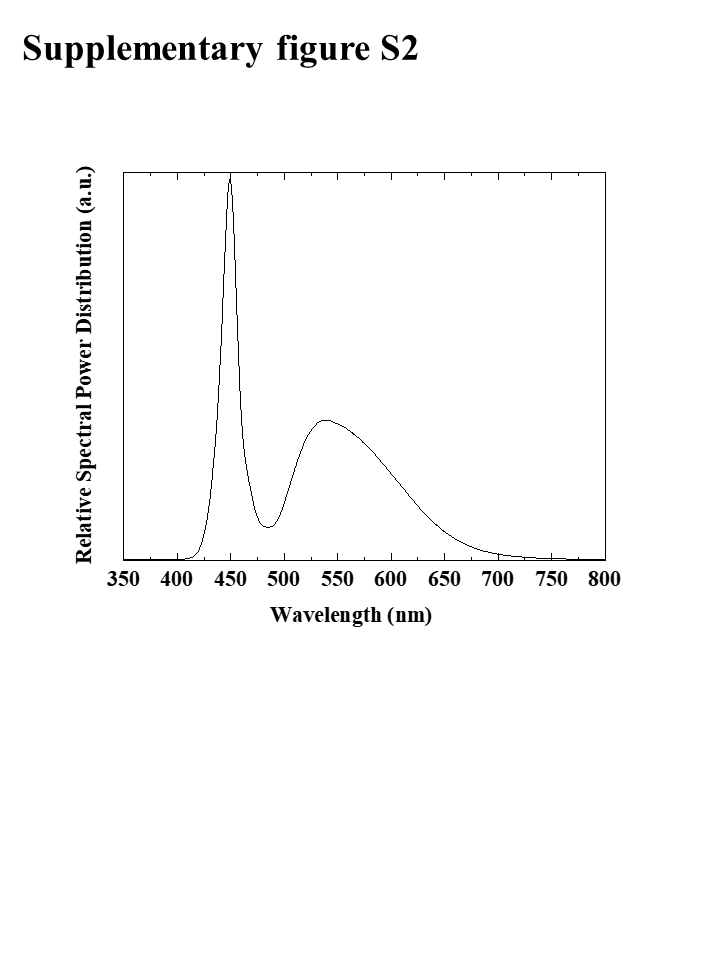

Supplement: Supplementary file 3 — Supplementary Information 2. [file 41598_2021_90460_MOESM3_ESM.tif]
