# Supplementary material for: A conductive polymer nanowire including functional quantum dots generated via pulsed laser irradiation for high-sensitivity sensor applications
Source: Sci Rep. 2021 May 27;11:11203. doi: 10.1038/s41598-021-90460-8 (PMC8159946; doi:10.1038/s41598-021-90460-8)
Supplement: Supplementary file 4 — Supplementary Information 3. [file 41598_2021_90460_MOESM4_ESM.docx]

Title: A Conductive Polymer Nanowire Including Functional Quantum Dots Generated via Pulsed Laser Irradiation for High-Sensitivity Sensor Applications

**Michiko Sasaki^1^& Masahiro Goto^1^**

^1^International Center for Materials Nanoarchitectonics (MANA), National Institute for Materials Science, 1-2-1 Sengen, Tsukuba, Ibaraki 305-0047, Japan
Correspondence and requests for materials should be addressed to M.S. (email: [sasaki.michiko@nims.go.jp](mailto:sasaki.michiko@nims.go.jp)) or M.G. (email: [goto.masahiro@nims.go.jp](mailto:goto.masahiro@nims.go.jp))

Acknowledgments

The authors are grateful to Mr. Takeshi Sunaoshi, Mr. Hiroyuki Ito, and Mr. Kohei Soda of Hitach High-Technologies Co., Ltd. for collecting the SEM and EDX images and measuring the electrical properties of the QD-CPNWs. This study was supported by JSPS KAKENHI Grant number 24656058.

**Supplementary Movie 1.** Time-resolved shadowgraphy movie of generation process of a PEDOT-PEG PNW including CdSe/ZnS core–shell QDs (QD-CPNW). QD-CPNW was generated from the polymer film surface by laser irradiation through the borosilicate glass substrate. The high-speed video camera recorded the shape of the QD-CPNW as a shadowgraphy movie. A timing generator was used for setting the time delays for the QD-CPNW generation laser, the flashlight, and high-speed video camera recording.

**Supplementary figure 1.** Comparison between the *I*–*V* curves of the CPNWs and QDs for different samples in the presence and absence of light irradiation: (a) without light, (b) with light.

**Supplementary figure 2.** The relative spectral power distribution of a light-emitting diode using irradiation for the CPNWs.
